# Premature Vertebral Mineralization in *hmx1*-Mutant Zebrafish

**DOI:** 10.3390/cells11071088

**Published:** 2022-03-24

**Authors:** Younes El Fersioui, Gaëtan Pinton, Nathalie Allaman-Pillet, Daniel F. Schorderet

**Affiliations:** 1 IRO—Institute for Research in Ophthalmology, 1950 Sion, Switzerland; gaetan.pinton@irovision.ch (G.P.); nathalie.allaman@irovision.ch (N.A.-P.); daniel.schorderet@hin.ch (D.F.S.); 2 Jules-Gonin Eye Hospital, Unit of Gene Therapy and Stem Cell Biology, 1004 Lausanne, Switzerland; 3 Faculty of Life Sciences, Swiss Federal Institute of Technology (EPFL), 1015 Lausanne, Switzerland; 4 Faculty of Biology and Medicine, University of Lausanne, 1011 Lausanne, Switzerland

**Keywords:** bone, vertebrae, zebrafish, *bmp2b*, *bmp4*, *noggin1*, *chordin*

## Abstract

H6 family homeobox 1 (HMX1) regulates multiple aspects of craniofacial development, and mutations in *HMX1* are linked to an ocular defect termed oculoauricular syndrome of Schorderet–Munier–Franceschetti (OAS) (MIM #612109). Recently, additional altered orofacial features have been reported, including short mandibular rami, asymmetry of the jaws, and altered premaxilla. We found that in two mutant zebrafish lines termed *hmx1^mut10^* and *hmx1^mut150^*, precocious mineralization of the proximal vertebrae occurred. *Zebrafish hmx1^mut10^* and *hmx1^mut150^* report mutations in the SD1 and HD domains, which are essential for dimerization and activity of *hmx1*. In *hmx1^mut10^*, the bone morphogenetic protein (BMP) antagonists chordin and noggin1 were downregulated, while bmp2b and bmp4 were highly expressed and specifically localized to the dorsal region prior to the initiation of the osteogenic process. The osteogenic promoters *runx2b* and *spp1* were also upregulated. Supplementation with DMH1—an inhibitor of the BMP signaling pathway—at the specific stage in which *bmp2b* and *bmp4* are highly expressed resulted in reduced vertebral mineralization, resembling the wildtype mineralization progress of the axial skeleton. These results point to a possible role of *hmx1* as part of a complex gene network that inhibits *bmp2b* and *bmp4* in the dorsal region, thus regulating early axial skeleton development.

## 1. Introduction

The oculoauricular syndrome of Schorderet–Munier–Franceschetti (OAS) (MIM:612109) is caused by defects associated with mutations in the *HMX1* transcription factor [1]. *HMX1*, belonging to a homeobox (*HMX*) family of transcription factors, presents a phylogenetically conserved 60-amino-acid homeobox domain [2]. OAS patients report aberrant orofacial development resulting in short mandibular rami, asymmetry of the jaws, and altered premaxilla [3].

In zebrafish, *hmx1*-deficient embryos exhibit increased apoptosis in the eyes and brain, in addition to a delayed withdrawal of retinal progenitors from the cell cycle [4]. The “*Dmbo*” mouse model showed malformations of the squamous temporal bone and hyperplasia of the gonial bone, and failed to develop somatosensory neurons in the geniculate ganglion [5,6].

The majority of the craniofacial bones are formed via direct ossification, while the cranial base, mammal limbs, and axial skeleton structures are formed via endochondral ossification—a process whereby a cartilage template is replaced by bone [7,8].

The dual process of ossification is conserved in teleost fish, including zebrafish. As early as 3 days post-fertilization (dpf) the cranial cartilage starts forming, followed by perichondral bone elements building up on the existing cartilage structures [9,10,11]. Within the same time window, the first bone elements—including the opercle, cleithrum, mandible, and maxilla—form via direct ossification [12,13]. Teleosts’ vertebral column is formed through direct mineralization of the notochord, and occurs progressively in a craniocaudal direction [14].

BMP gradient is essential for dorsoventral patterning. and is highly modulated through different mechanisms, including extramembranous regulation. *Chordin*, *follistatin*, and *noggin* negatively regulate BMP signaling by binding BMP ligands and preventing their interaction with transducer receptors localized on the cell membranes of target cells [15,16,17]. BMP expression, following dorsoventral axis formation and regulation, is required for the osteogenic process to proceed [18].

In zebrafish, BMP signaling is crucial between 48 and 72 h post-fertilization (hpf) for head cartilage development and bone mineralization [19].

The r*unx2a* and *runx2b* orthologs initiate both endochondral and intramembranous ossification [20,21,22]. BMP–*Runx2* interaction initiates osteoblast differentiation and drives the onset of genes involved in bone extracellular matrix deposition, such as bone gamma-carboxyglutamate (Gla) protein (*Bglap*) and secreted phosphoprotein 1 (*Spp1*) [23,24,25,26].

Disruption of BMP signaling is associated with several human diseases [27], and deletion of *BMP2* and *BMP4* or *BMP2* alone results in a severe chondrodysplastic phenotype [28], while craniofacial defects were reported in conditional knockout mice lacking the BMP type I receptor Alk2 [29]. In zebrafish, a mutation in *bmp1a* resulted in mature bone with a higher mineral content [30].

Despite the recent reports on the phenotypic defects associated with *HMX1* mutation, not much is known about its role in transcriptional regulation. HMX1 binds preferentially to the consensus sequence 5′-CAAGTG-3′ located in the promoter region of target genes, and acts as a transcriptional regulator [31]. We previously designed a predictive promoter model [32] and generated two zebrafish models—*hmx1^mut10^* and *hmx1^mut150^* mutant lines—to screen and identify potential hmx1 target genes [33]. The *hmx1^mut10^* and *hmx1^mut150^* mutant lines carry mutations in the SD1 and homeodomain domains, which are implicated in the dimerization and activity of *hmx1* [34]. Embryos collected from both mutant zebrafish lines presented eye-related defects in a similar manner to the phenotype observed in human patients, and at present, while there a growing number of articles focus on *HMX1*-related ocular defects, not much is known about its role during bone formation. With the present work, we aimed at investigating the contribution of *hmx1* during skeletogenesis in zebrafish. 

## 2. Material and Methods

### 2.1. Zebrafish and Mouse Maintenance and Breeding

All animal procedures were carried out in accordance with the policies established by the ARVO (Association for Research in Vision and Ophthalmology) Statement for the Use of Animals, and followed FELASA (Federation of European Laboratory Animal Science Associations) recommendations on the use of zebrafish [35]. Experiments were approved by the Veterinary Service of the State of Valais (Switzerland). Zebrafish (Danio rerio) were maintained in a 14 h/10 h light/dark cycle; embryos were kept at 28.5 °C in E3 medium [35]. All embryos at desired stages were kept in Danieau’s solution with 0.003% 1-phenyl-2-thiourea (Sigma, Buchs, Switzerland) to suppress pigmentation. The zebrafish mutant lines *hmx1^mut10^* and *hmx1^mut150^* were previously generated and described [33]. Briefly, *hmx1^mut10^* zebrafish have a frameshift mutation that generates a termination codon in the SD1 domain, while mutant *hmx1^mut150^* has an indel mutation that replaces the HD domain; dimerization of SD1 and HD is necessary for the proper function of *hmx1*.

### 2.2. RNA Extraction, cDNA Synthesis, and RT-PCR

Wildtype and *hmx1*-knockout embryos at different stages up to 5 dpf were collected and euthanatized. Thirty embryos were obtained from different breeding groups and pooled together. All experiments were repeated three times.

Total RNA was extracted from samples using the RNeasy Micro Kit (Qiagen; Hombrechtikon, Switzerland). Primers specific to the selected genes were designed (Supplementary Table S1), and first-strand cDNA synthesis using the AffinityScript™ Multiple Temperature Reverse Transcriptase Kit (Agilent; Basel, Switzerland) was performed according to the manufacturer’s protocol. cDNA was generated (GoScript Reverse Transcriptase System; Promega; Dübendorf, Switzerland), and real-time RT-PCR (FastStart SYBR Green Master Roche; Rotkreuz, Switzerland) was performed following standard protocols. Gene expression changes were determined using the 2^–ΔΔCt^ method; relative values were normalized with β-actin. 

### 2.3. Statistical Analysis

Averages of the different experiments were expressed as the mean ± SEM.; Student’s *t*-test was used to express the significance of differences between two groups. Significance was set at 0.05.

### 2.4. Alcian Blue and Alizarin Red Staining

Zebrafish embryos at different embryonic stages were euthanatized, rinsed with PBS, and fixed with 4% paraformaldehyde overnight. Specimens were preserved in 100% methanol.

Staining for bone was performed with 0.1% alizarin red (Fluka; Buchs, Switzerland) and 0.03% KOH in ddH20 for 30 min. After the washing step, zebrafish embryos were conserved in 1% KOH/50% glycerol.

Staining for cartilage was performed using alcian blue solution (0.1% alcian blue, 1% concentrated hydrochloric acid, 70% ethanol) (Sigma, CAS No: 33864-99-2; Buchs, Switzerland) for 30 min. Embryos were washed and cleared in acidic ethanol (5% hydrochloric acid, 70% ethanol); after dehydration in an ethanol series, they were stored in glycerol. Pictures were taken using an Olympus DP71 camera.

### 2.5. Whole-Mount in Situ Hybridization

Whole-mount staining was performed as previously described [34]. Sense and antisense probes for *bmp2b*, *bmp4*, and *runx2b* were synthetized by transcription of cDNA clones with T7 and SP6 RNA polymerase, using digoxigenin labeling mix. Zebrafish embryos were fixed at 2 dpf in 4% paraformaldehyde. The hybridization reaction was carried out at 68 °C for 14–18 h. Incubation and washing were performed using the BioLane HTI system (Hölle & Hüttner, Tubingen, Germany).

### 2.6. DMH1 Treatment

For BMP signaling inhibition, 10 mM DMH1 stock solution (Sigma, CAS. No: 1206711-16-1) was diluted in DMSO. Embryos at 2 dpf were placed in a 6-well plate with the inhibitor diluted in E3 medium at 50 μM or 100 μM overnight; 0.1% DMSO in E3 was used as a control. DMH1 solution was replaced with E3 medium, and at the desired stage the embryos were collected and fixed. All experiments were performed on 30 individuals per zebrafish group and repeated at least 3 times.

## 3. Results

### 3.1. Hmx1^mut10^ Zebrafish Present Premature Vertebrae Mineralization

**Figure 1:**
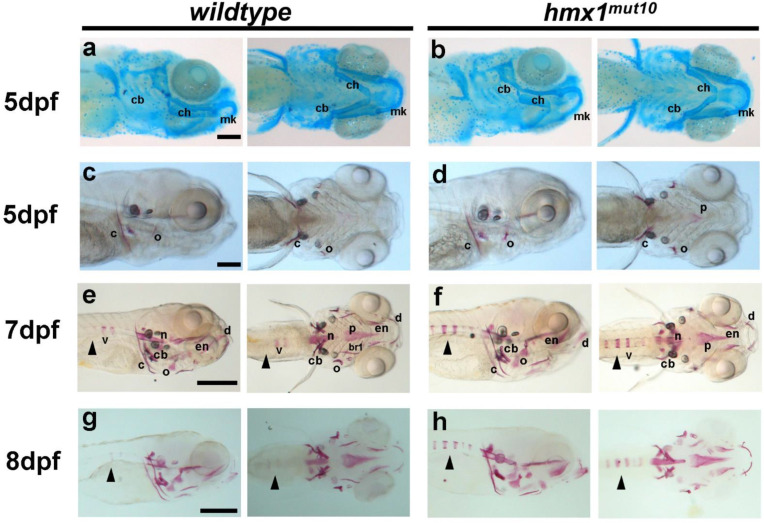
*Hmx1^mut10^* zebrafish embryos develop early mineralized vertebrae: Cartilage structures stained with alcian blue in wildtype and *hmx1^mut10^* embryos (**a**,**b**) at 5 dpf; no morphological differences in the developing cranial cartilage structures were detected. Cranial bone structures stained with alizarin red in wildtype and *hmx1^mut150^* at 5 dpf (**c**,**d**); wildtype and *hmx1^mut10^* present regular development of bone structures. At 7 dpf and 8 dpf, alizarin red staining detected an early and progressive mineralization of the vertebrae in *hmx1^mut10^* (**f**,**h**) in comparison to wildtype zebrafish (**e**,**g**). cb, ceratobranchial pairs; ch, ceratohyal; mk, Meckel’s cartilage; v, vertebrae; c, cleithrum; n, notochord; cb, ceratobranchial 5; en, entopterygoid; o, opercle; d, dentary. Black arrowhead: early mineralization of the vertebrae in *hmx1^mut10^* (**f**,**h**). Bar, (**a**–**c**) 250 μm; (**e**–**g**) 500 μm.

Human patients carrying *HMX1* mutations presented defects related to bone development [3]; therefore, we asked whether the mutations generated in *hmx1* would affect cranial cartilage and bone development. We performed alcian blue and alizarin red staining on wildtype and *hmx1^mut10^* embryos. At 5 dpf in hmx1^mut10^, all cranial cartilaginous elements were present and unaffected in their development. Meckel’s cartilage, ceratohyal, and branchial arches were formed and fully developed in both wildtype and *hmx1^mut10^* embryos (Figure 1a,b). Head skeleton formation was initiated subsequently to cartilage formation starting at 3 dpf, and at 5 dpf cranial bones were generally fully formed. Structures formed via intramembranous ossification—such as the cleithrum, anterior notochord, and operculum—were formed and visible at 5 dpf in wildtype and *hmx1^mut10^* embryos. A morphological inspection showed that there were no differences in bone size, shape, or localization (Figure 1c,d).

In wildtype zebrafish, following the formation of the anterior notochord, mineralization of the vertebrae progresses towards the caudal region, with 70% of the embryos presenting one mineralized vertebra, while the remaining assessed embryos either presented two alizarin-red-stained vertebrae or none at 7 dpf (Figure 1e,f). At the same embryonic stage, *hmx1^mut10^* zebrafish presented a precocious mineralization, with several additional vertebrae positive for alizarin red; at 8 dpf, the differential mineralization pattern was still maintained, with approximately 80% of embryos presenting five mineralized vertebrae (Figure 1g,h). 

### 3.2. Hmx1^mut150^ Zebrafish Recapitulate Premature Vertebral Mineralization Similarly to Hmx1^mut10^ Mutants

**Figure 2:**
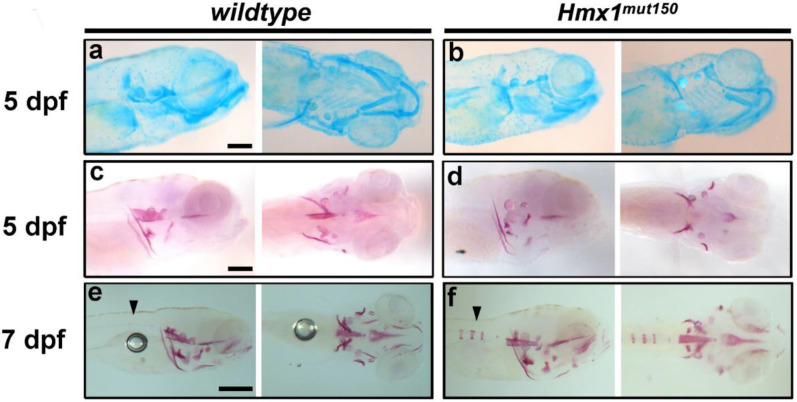
Precocious vertebral bone development in *hmx1^mut150^* zebrafish embryos: Cartilage structures stained with alcian blue in wildtype and *hmx1^mut150^* embryos (**a**,**b**) at 5 dpf; the developing cranial cartilage structures develop properly in *hmx1*-mutant embryos. Cranial bone structures stained with alizarin red in wildtype and *hmx1^mut150^* at 5 and 7 dpf (**c**,**f**). Wildtype and *hmx1^mut150^* cranial bones developed regularly at 5 dpf (**c**,**d**), while at 7 dpf early mineralization of the vertebrae was detected in *hmx1^mut150^,* similarly to *hmx1^mut10^* embryos (**e**,**f**). Bar, (**a**–**c**) 250 μm; (**e**–**f**) 500 μm.

*Hmx1* presents three conserved regions: the SD1, SD2, and HD domains. *Hmx1^mut150^* zebrafish carry an indel mutation that replaces the HD domain. Dimerization of HD and SD1 is necessary for hmx1 activity. Alizarin red staining at 5 dpf of *hmx1^mut150^* embryos showed that at 5 dpf both cartilage (Figure 2a,b) and bone (Figure 2c,d) structures were formed. At 7 dpf, *hmx1^mut150^—*similarly to *hmx1^mut10^—*presented an early mineralization pattern of the first cranial vertebrae (Figure 2e,f).

### 3.3. Osteogenic Signaling during Early Bone Development in Wildtype and Hmx1^mut10^ Zebrafish

We aimed at describing the time course of early osteogenesis by profiling bone-related genes. We focused on genes encoding for BMP antagonists and BMPs themselves, in addition to a master transcription factor and a gene involved in osteoblast function and mineralization.

**Figure 3:**
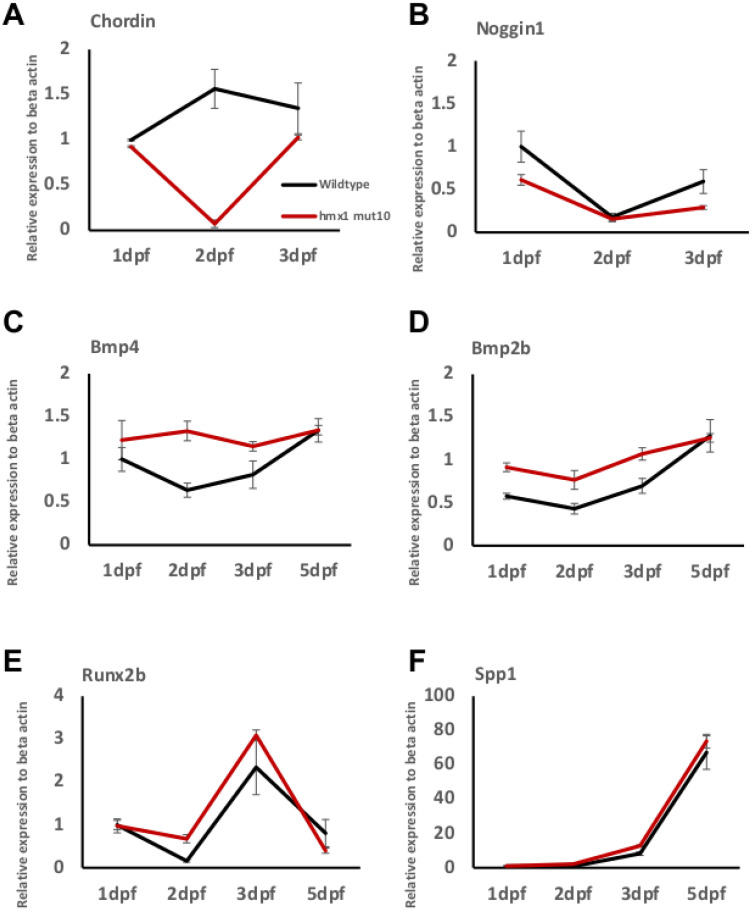
*Hmx1^mut10^* embryos present enhanced expression of osteogenic factors: RT-PCR analysis of the BMP antagonist *chordin* and noggin1 relative to β-actin, the osteogenic inducers *bmp2b* and *bmp4*, and the osteogenic markers *runx2b* and *spp1*. In *hmx1^mut10^* embryos, *chordin* expression is reduced at 2 dpf (**A**), while *noggin1* expression is lower at 1 dpf and 3 dpf (**B**). *Bmp2b* and *bmp4* transcripts are significantly different in *hmx1^mut10^* embryos when compared to wildtype zebrafish from 1 dpf to 3 dpf (**C**,**D**). The osteogenic markers *runx2b* and *spp1* are differentially expressed, and are higher in *hmx1^mut10^* at 2 dpf and 3 dpf, respectively (**E**,**F**). Data are expressed as mean of three or more experiments.

Total RNA was isolated from wildtype and mutant zebrafish embryos collected from 1 dpf to 5 dpf, and RT-PCR analysis was performed. During early development, the antagonists *chordin* and *noggin* are expressed in the dorsal region and, thus, restrict bmp activity in the dorsal region [16,36]. RT-PCR analysis of *chordin* indicated that the antagonist expression did not alter from 1 dpf to 3 dpf in wildtype embryos, while in *hmx1^mut10^, chordin* expression was greatly reduced at 2 dpf (Figure 3A). *Noggin1* expression, on the other hand, was significantly reduced at 1 dpf and 3 dpf in *hmx1^mut10^* in comparison to wildtype embryos (Figure 3B).

The osteogenic differentiation process is initiated upon expression of BMP factors—namely, *bmp2* and *bmp4*—followed by the specific early marker of osteoprogenitor cells *runx2b*. Starting at 1 dpf, and continuously until 3 dpf, *bmp2b* and *bmp4* transcripts were significantly greater in *hmx1^mut10^* compared to wildtype zebrafish; at 5 dpf, *bmp2b* and *bmp4* transcript quantities were stabilized at the same expression level in both wildtype and *hmx1^mut10^* zebrafish (Figure 3C,D). In regard to the expression of *runx2b*, the transcription factor was differentially expressed, and presented a higher RNA level at 2 dpf; *spp1* expression followed the expression of *runx2b*, and it was significantly higher at 3 dpf in *hmx1^mut10^* (Figure 3E,F). Finally, *runx2b* and *spp1* expression patterns detected in mutant zebrafish resembled the pattern observed in wildtype embryos.

### 3.4. Bmp2b and bmp4 Are Expressed in the Dorsal Region in Hmx1^mut10^ Embryos

**Figure 4:**
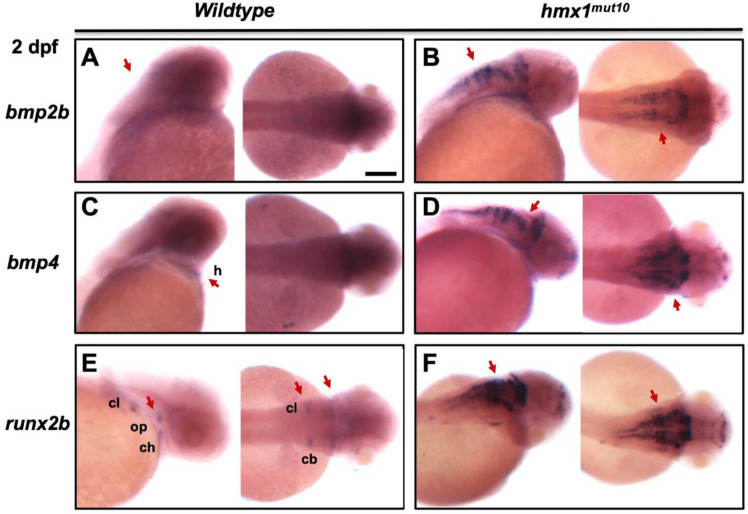
In situ hybridization detected bmp2b and bmp4 the dorsal region in Hmx1^mut10^ embryos: In situ hybridization in wildtype and *hmx1^mut10^* embryos at 2 dpf. Staining for *bmp2b*, *bmp4*, and *runx2b.* No signal was detected for *bmp2b* and *bmp4* in the dorsal region of wildtype zebrafish, while *bmp4* was detected in the heart region ((**A**,**C**) red arrows). In *hmx1^mut10^*, *bmp2b* and *bmp4* are expressed in the dorsal region (**B**,**D**). *Runx2b* in wildtype embryos at 2 dpf is expressed in the bone-forming regions (**E**). In *hmx1^mut10^* embryos, *runx2b* is expressed in the dorsal region, and presents a similar pattern to *bmp2b* and *bmp4* (**F**). Cl, cleithrum; ch, ceratohyal; cb, ceratobranchial; h, heart; op, opercle. Bar, A–F 100 μm.

Given the increased expression of skeletal-associated genes during early development, we performed whole-mount in situ hybridization to track the localization of *bmp2b*, *bmp4*, and the osteogenic transcription factor *runx2b* (Supplementary Figure S1). In wildtype embryos, *bmp2b* and *bmp4* expression is inhibited by the antagonists *noggin* and *chordin* [37,38] in the dorsal region during early development. We found that prior to the initiation of the osteogenic process, at 2 dpf, there was no signal detected for *bmp2b* and *bmp4* in the wildtype dorsal region (Figure 4a,c). Inspection of *hmx1^mut10^* evidenced strong and localized signals for *bmp2b* and *bmp4* in the dorsal region in the correspondence of the hind–midbrain, along with an increased signal in specific bone-forming domains of the cranial frontal region (Figure 4b,d). *Bmp2* induces *runx2b* expression; therefore, we analyzed the expression pattern of *runx2b* in zebrafish embryos at the same stage. In wildtype embryos, as expected, *runx2b* at 2 dpf was expressed in the opercle, ceratohyal, Meckel’s cartilage, ceratobranchial cartilage, and cleithrum (Figure 4e); *hmx1^mut10^* presented a spread and stronger signal for *runx2b* in the dorsal region at the same stage. In situ hybridization for *runx2b* presented a pattern identical to *bmp2b* and *bmp4* expression (Figure 4f).

### 3.5. Inhibition of bmp Signaling at 2 dpf Reduced the Progression of Vertebral Mineralization in Hmx1^mut10^ Embryos

**Figure 5:**
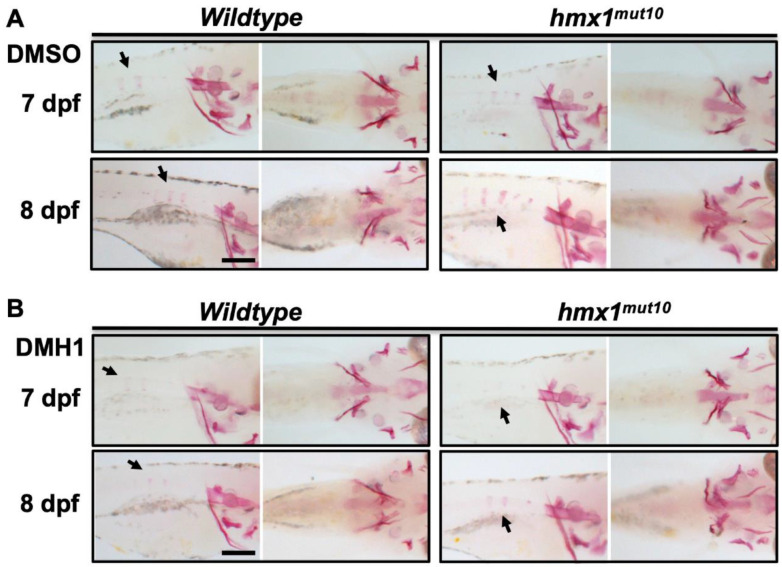
DMH1-mediated inhibition of BMP signaling reduces the progression of vertebral mineralization**:** Wildtype and *hmx1^mut10^* embryos were treated with 50 μM DMH1 at 2 dpf and placed in E3 medium at 3 dpf. Alizarin red staining to detect mineralized structures was performed at 7 and 8 dpf (left, lateral view, right ventral view). Wildtype and *hmx1^mut10^* control embryos treated with DMSO (**A**). Precocious vertebral mineralization was inhibited in DMH1-treated *hmx1^mut10^* embryos at 7 dpf (**B**). At 8 dpf, treated *hmx1^mut10^* embryos still presented a vertebral mineralization pattern similar to wildtype embryos (**B**). Bar, (**A**,**B**) 500 μm.

Mutant embryos presenting precocious mineralization of the vertebrae showed localized BMP signaling in the dorsal region during early embryonic development. To confirm the involvement of *bmp2* and *bmp4* factors in the developing vertebrae in mutant embryos, we investigated the effects of DMH1—an inhibitor of BMP-specific signaling [39]. Overnight treatment with 50 μM or 100 μM concentrations of DMH1 was performed at 2 dpf. Wildtype and *hmx1^mut10^* were collected at 7 and 8 dpf; alizarin red staining was performed to detect cranial and vertebral mineralization. Wildtype embryos treated with 100 mM DMH1 were viable, and showed reduced mineralization in the cranial region (Supplementary Figure S2A(a,c),B(a–d)), while *hmx1^mut10^* reported aberrant development, including cardiac edema, and resulting in their failure to grow (Supplementary Figure S2A(b–d)). Alizarin red staining of wildtype embryos treated with 50 μM DMH1 at 8 dpf showed that the treatment did not lead to reduced cranial mineralization, and that vertebral bone development continued to progress (Figure 5B). Cranial bone development in *hmx1^mut10^* treated with 50 μM DHM1 was unaffected, while the precocious vertebral mineralization was inhibited, resulting in a mineralization pattern similar to that of wildtype embryos (Figure 5B). Wildtype and *hmx1^mut10^* embryos treated with DMSO did not show altered development or aberrant bone development.

## 4. Discussion

The *HMX* gene family is involved in the development of the sensory organs, and mutations in *HMX1* result in a set of defects affecting the development of the retina, along with other congenital eye-related defects [1,40]. Patients carrying mutations of *HMX1* presented an additional subset of defective maxillomandibular structures and spina bifida [3]. This indicates a possible additional role for *HMX1* in the regulation of several developmental processes specific to craniofacial and axial skeleton formation.

We have previously generated two mutated zebrafish lines for *hmx1; hmx1^mut10^* and *hmx1^mut150^* carry mutations affecting the SD1 and homeodomain domains—both essential for an active dimeric hmx1 protein [34]. Inspection of *hmx1^mut10^* at 5 dpf indicated that both maturation of cartilaginous structures and mineralization of the cranial bones were unaffected (Figure 1a–d). In wildtype embryos, vertebral development initiated in a timely manner, while mineralization of the axial skeleton in *hmx1^mut10^* at the same stages was accelerated, with several vertebrae presenting early mineralization at 7 and 8 dpf (Figure 1e–h). *Hmx1^mut10^* and *hmx1^mut150^* zebrafish lines both showed early mineralization of the vertebrae, indicating that mutations affecting either the SD1 or homeodomain domains result in the same altered osteogenic phenotype.

*Noggin* [41] and *chordin* activity is required for proper axial skeleton development [16,42]. Whole-embryo RT-PCR analysis showed reduced expression of *chordin* at 1 dpf and 3 dpf, and of *noggin* at 2 dpf, in *hmx1^mut10^* embryos (Figure 3A–B). Short-term expression of BMP-2 is necessary to induce bone formation [43] by upregulating the expression of the osteogenic markers *runxb2* and *spp1* [44], and loss of both BMP-2 and BMP-4 results in severe impairment of osteogenesis [45]. *Bmp2b* and *bmp4* were highly expressed in *hmx1^mut10^* embryos (Figure 3C–D), followed by an increased sequential expression of *runxb2* and *spp1* at 2 dpf and 3 dpf when compared to wildtype embryos (Figure 3E–F).

In situ hybridization showed a strong and broad signal for *bmp2b* and *bmp4* in the dorsal region in *hmx1^mut10^* embryos. *Runx2b* presents a dorsal pattern similar to those of *bmp2b* and *bmp4*, whereas in wildtype embryos at 2 dpf it was specifically localized to the ventral osteogenic sites (Figure 3).

Treatment with DMH1—a highly selective BMP signaling inhibitor widely used in zebrafish models—limited vertebral mineralization in *hmx1^mut10^* zebrafish, with no effect on cranial bone development in either *hmx1^mut10^* or wildtype zebrafish (Figure 4), confirming that early mineralization of the vertebrae occurred in response to the increased *bmp2b* and *bmp4* transcripts [46,47,48]. Noggin and DMH1 have been shown to have a similar effect in regulating the expression of a set of genes involved in neurogenesis [49].

The question of whether hmx1 directly regulates *bmp2b* and *bmp4* could be assessed by employing the heat-shock zebrafish model that we utilized to validate potential hmx1 target genes in vivo [34]; however, we do not believe that this is the case. By accessing the Eukaryotic Promoter Database (https://epd.epfl.ch; accessed 12 December 2020), and using the predictive promoter model, we screened *bmp2b* and *bmp4*, as well the *noggin1* and *chordin* regions upstream of the transcription starting site. We did not identify CAAGTG binding sites in *bmp2b* and *bmp4*, but several clusters of CAAG binding sites common to humans, mice, and zebrafish were located on the potential promoter region of *noggin1* and *chordin*, (Supplementary Figure S3). It appears more plausible that hmx1 induces *bmp2b* and *bmp4* expression through an indirect mechanism.

Previously, we identified *uhrf1* as an hmx1 target gene, and it has been implicated in the regulation of several developmental and homeostatic processes related to zebrafish development. In situ hybridization in *hmx1* transgenic and mutant zebrafish showed that *uhrf1* expression is modulated in the hindbrain, eye region, and branchial arches [33]. Potentially, uhrf1 could change the methylation pattern of *bmp2b* and *bmp4* and, therefore, induce gene transcription. Reductions in *noggin1* and *chordin* expression could follow as part of a feedback mechanism. In this view, we could analyze the methylome patterns of *bmp2b* and *bmp4* in the dorsal region at critical stages for axial skeletogenesis.

Another potential indirect mechanism could be linked to the dorsoventral polarization process; it is well known that *bmp2b* and *bmp4* are expressed following a gradient pattern across the dorsoventral axis. *Noggin1* and *chordin* are expressed dorsally and, therefore, inhibit BMP expression. Given that potential *noggin1* and *chordin* promoter regions present several hmx1 binding sites, and that we observed a reduced expression in *hmx1^mut10^* zebrafish, we could assume that in wildtype zebrafish hmx1 contributes to regulating *noggin1* and *chordin* expression, consequently modulating *bmp2b* and *bmp4* expression in the dorsal region. In the mutated *hmx1* background, *noggin1* and *chordin* expression is reduced, thus inducing precocious *bmp2b*, *bmp4*, and *runx2b* expression and initiating axial bone formation.

Our work indicates that *hmx1* could be a modulator contributing to the maintenance of the BMP gradient in the dorsal region during axial bone development. In vitro studies of hmx1 activity on the *chordin* and *noggin1* promoter regions, coupled with *hmx1* heat-shock experiments, could elucidate the contribution of *hmx1* during axial development.

## Data Availability

All data analyzed during this study are included in this article or Supplementary Materials.

## Supplementary Materials

The following supporting information can be downloaded at: https://www.mdpi.com/article/10.3390/cells11071088/s1, Figure S1: Alcian blue and alizarin red staining of Wildtype and *hmx1^mut150^* embryos; Figure S2: DMH1 treatment of Wildtype and *hmx1^mut150^* embryos at 2 dpf; Figure S3: Schematic representation of HMX1 predicted promoter regions in human, zebrafish and mouse; Table S1: Primers.
