# Supplementary material for: Premature Vertebral Mineralization in hmx1-Mutant Zebrafish
Source: Cells. 2022 Mar 24;11(7):1088. doi: 10.3390/cells11071088 (PMC8997757; doi:10.3390/cells11071088)
Supplement: Supplementary file 1 [file cells-11-01088-s001.zip › cells-1615549-supplementary.pdf]

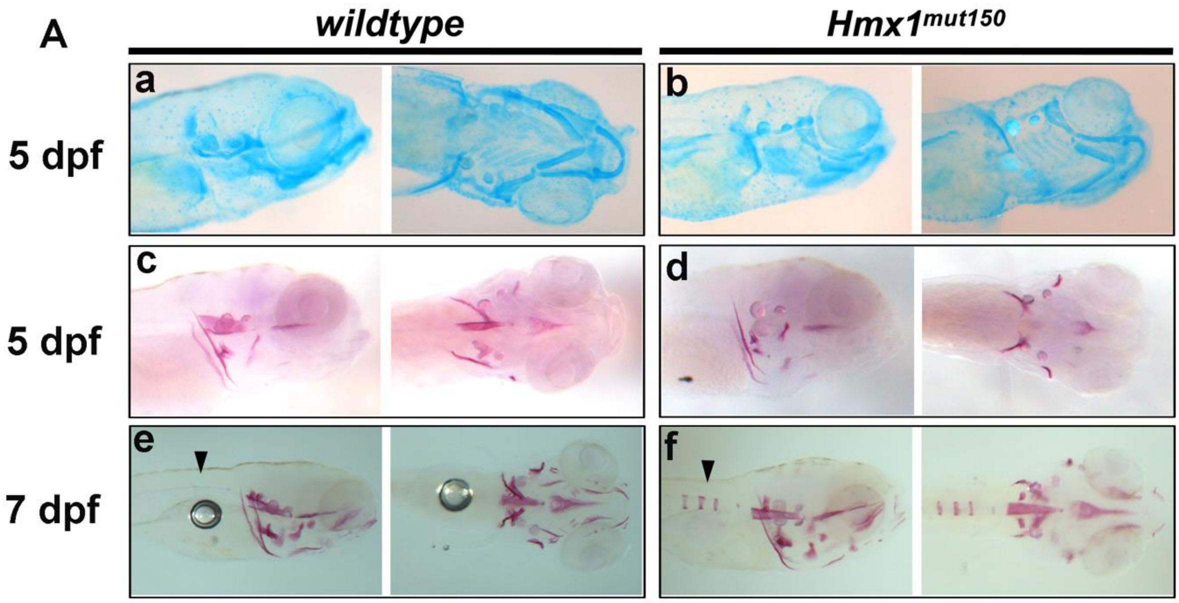

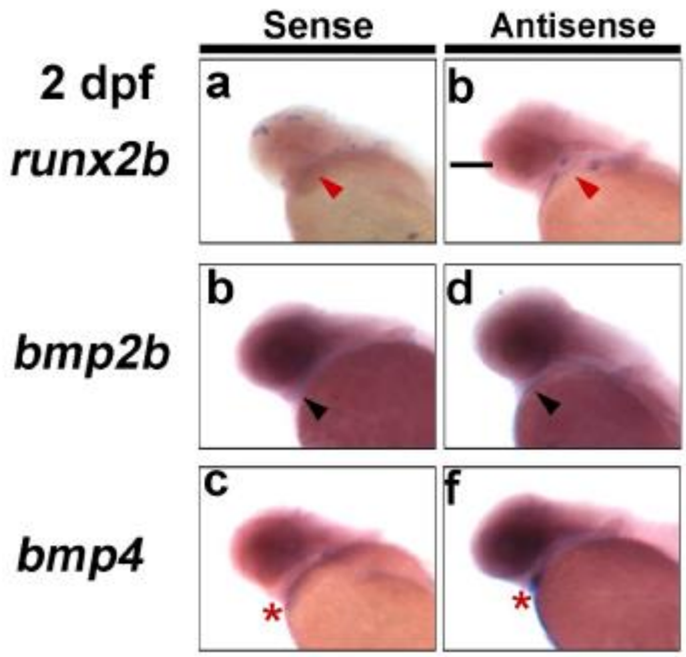

Supplementary figure S1: Alcian blue and alizarin red staining of Wildtype and *hmx1*^{mut150} embryos. *hmx1*^{mut150} develop proper cranial cartilage structures in a similar manner to wildtype embryos (Supp. A, a-b). *Hmx1*^{mut150} stained with alizarin red to visualize cranial bones, do not show altered skeletogenesis at 5 dpf (Supp. A, c-d), while at 7 dpf exhibit a precocious vertebral mineralization in comparison to wildtype (Supp. A, e-f). In situ hybridization performed with sense (Supp. 1B, a,c,e) and antisense probes (Supp. 1B, b,d,f). *Runx2b* is detected in the branchial region (red arrow head); *bmp2b* (black arrow head) and *bmp4* (asterisk) are detected in the cardiac region.

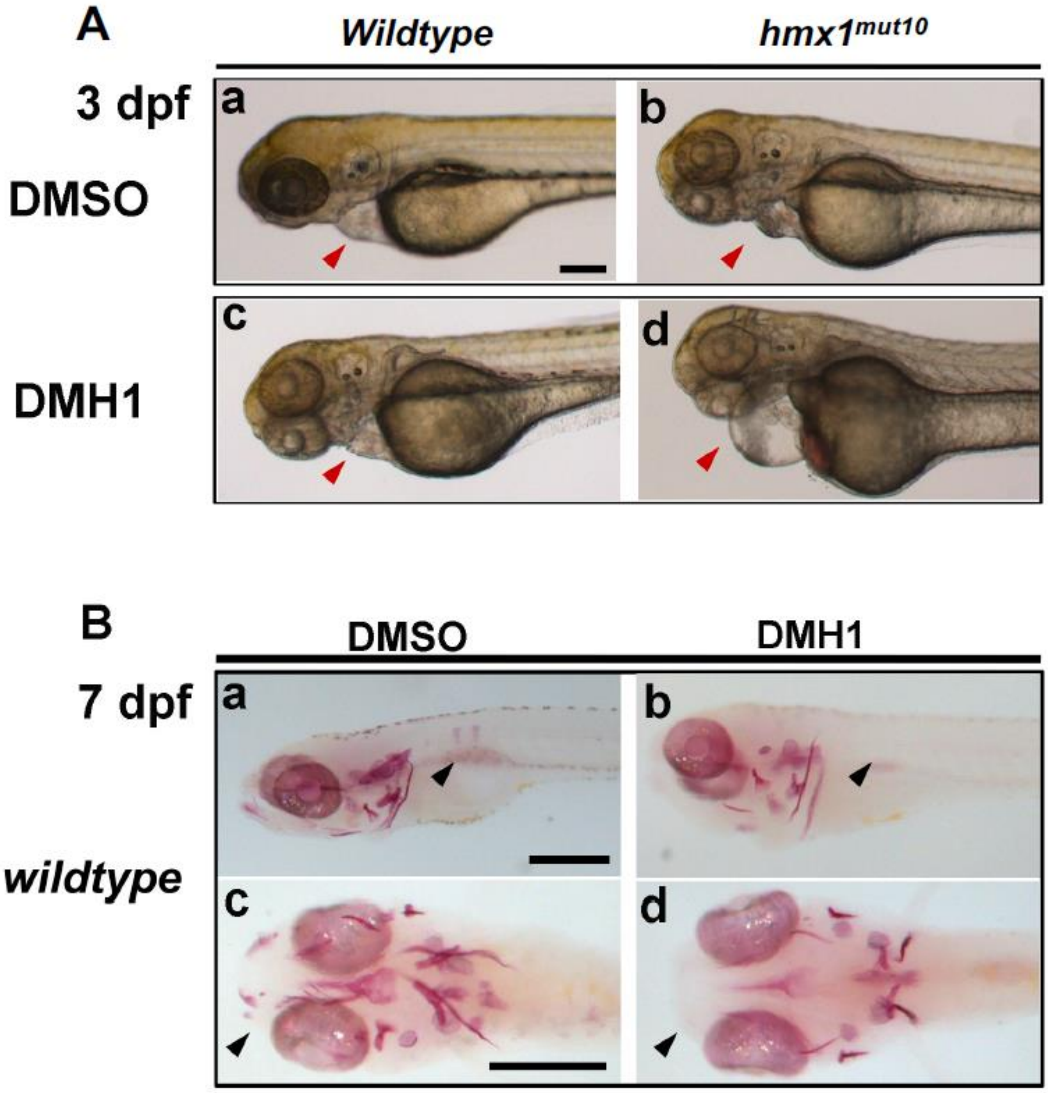

Supplementary figure S2: DMH1 treatment of Wildtype and *hmx1*^{mut10} embryos at 2 dpf. Wildtype and *hmx1*^{mut10} embryos were treated with DMH1 100 µM at 2 dpf, collected at 3 dpf and stained at 7 dpf with alizarin red staining. Morphological inspection of *hmx1*^{mut10} embryos at 3dpf showed that *hmx1* mutant embryos developed cardiac edema and failed to further develop when compared with *hmx1* control group treated with DMSO (Supp. 2A, b,d). Wildtype embryos treated with DMH1 100 µM did not show altered development at 3 dpf (Supp. 2A, a,c). Alizarin staining of wildtype zebrafish at 7 dpf shows that cranial bones as well as vertebral development were inhibited (Supp. 2B, b,d). wildtype treated with DMSO developed proper bone structures and vertebrae (Supp. 2B, a,c). *hmx1*^{mut10} embryos did not recover and were not collected at 7 dpf stage. Black arrow head indicate the missing vertebral and mandibular mineralization in DMH1 treated embryos (Supp. 2B, b,d) in comparison to control wildtype embryos treated with DMSO (Supp. 2B, a,c).

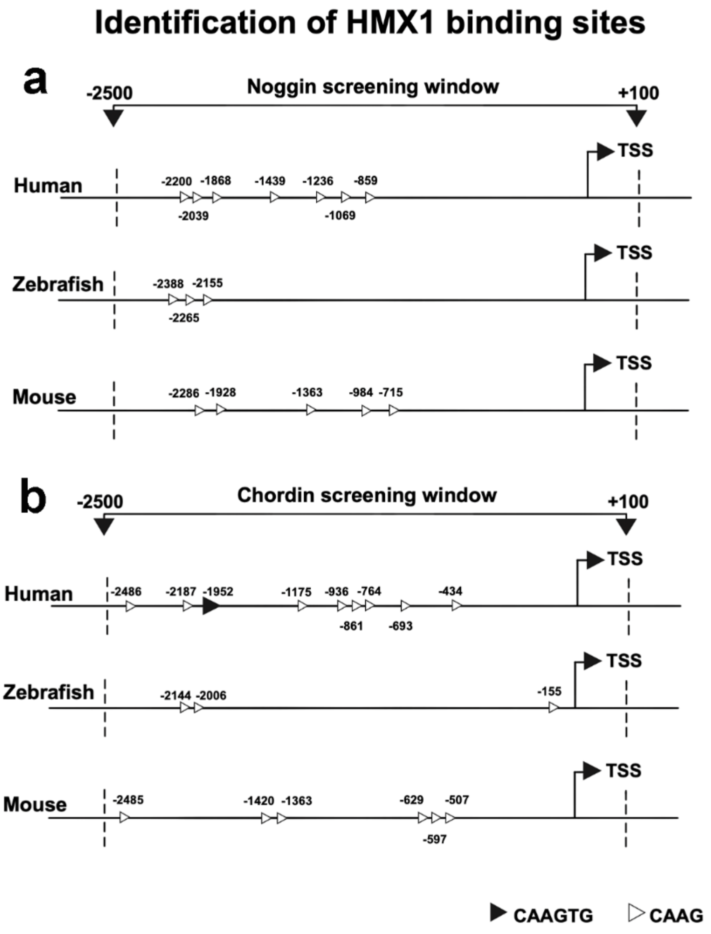

Supplementary figure S3: Schematic representation of HMX1 predicted promoter regions in human, zebrafish and mouse. Screening of HMX1 complete binding sites (CAAGTG) and minimal binding core (CAAG) located upstream of the transcription starting site of *noggin1* (Supp. 3, a) and *chordin* (Supp. 3, b).

Table S1: Primers

**In Situ**	**bmp2b-fw**	**CGAGATCGACCGACGGAAAT**	**bmp2b-rev**	**CCTCGAAAGCCTCTTCGTGAT**
In Situ	bmp4-fw	GGAGTGCTGTTCTCGAGTGT	bmp4-rev	GCGGGGAGATCCTTTTCCATT
In Situ	runx2b-fw	TGCCTTCTCATTGGAGGTGTAATA	runx2b-rev	CCACCGTGACCTTAATGGCT
RT-PCR	chordin-fw	CGCATCTGTGCACGGATCGAGA CRC	chordin-rev	CAGCCAAGAGTCTGTCACACCCGTC
RT-PCR	noggin1-fw	AAGCCAGCAAGAAGCTGAAG	noggin1-rev	GTTGATTTGGCCGGTTTG
RT-PCR	bmp2b-fw	GCACAAGTATGAACAAGAAGAG GC	bmp2b-rev	CCGAACATATTGAGCAAGCGTAG
RT-PCR	bmp4-fw	CCAACACCGTGAGAGGATTCC	bmp4-rev	TCCACAGCAAGGCCATGATTAG
RT-PCR	runx2b-fw	GTGGCCACTTACCACAGAGC	runx2b-rev	TCGGAGAGTCATCCAGCTT
RT-PCR	spp1-fw	CGCCACAGTCTTCTGTGTACC	spp1-rev	TTGAACAATTACAAGCTCTTCTGAG
